# Phytobezoar-Induced Mechanical Ileus and Incipient Intussusception: A Case Report

**DOI:** 10.3390/medicina59071227

**Published:** 2023-06-30

**Authors:** Christoforos S. Kosmidis, Chrysi Maria Mystakidou, Nikolaos Varsamis, Charilaos Koulouris, Christina Sevva, Konstantina Papadopoulou, Christina Michael, Nikolaos Iason Katsios, Vasiliki Theodorou, Petrina Miltiadous, Konstantinos Papadopoulos, Konstantinos Vlassopoulos, Katerina Zarampouka, Stylianos Mantalovas

**Affiliations:** 1 European Interbalkan Medical Center, 10 Asklipiou Street, 55535 Pylaia, Greece; 2 3rd Surgical Department, University General Hospital of Thessaloniki “AHEPA”, School of Medicine, Faculty of Health Sciences, Aristotle University of Thessaloniki, 1st St. Kiriakidi Street, 54621 Thessaloniki, Greece; 3 Medical School, Faculty of Health Sciences, Aristotle University of Thessaloniki, 54124 Thessaloniki, Greece; 4 1st Department of Internal Medicine, G. Papanikolaou General Hospital of Thessaloniki, 57010 Thessaloniki, Greece; 5 Medical School, Faculty of Health Sciences, University of Ioannina, 45110 Ioannina, Greece; 6 Pathology Department, Aristotle University of Thessaloniki, AHEPA University Hospital, 1st St. Kiriakidi Street, 54621 Thessaloniki, Greece

**Keywords:** phytobezoar, intestinal obstruction, ileus, intussusception

## Abstract

Phytobezoars constitute conglomerates of indigested plant fibers and are a rare cause of acute mechanical ileus. They exhibit an increased prevalence in the elderly population and people with specific predisposing conditions. Radiological imaging can often set a definitive diagnosis and dictate the optimal therapeutic approach, combined with the patient’s clinical status. An 81-year-old male presented with deteriorating clinical symptoms of intestinal obstruction, and an exploratory laparotomy was performed following inconclusive radiological findings; multiple phytobezoars and incipient intussusception were revealed intraoperatively. A patient’s medical history can often raise clinical suspicion of phytobezoars. However, a careful etiological investigation is imperative in all cases of mechanical ileus in advanced ages; early detection and dissolution of phytobezoars, when applicable, can reduce the need for surgical interventions.

## 1. Introduction

The term “bezoar” was officially introduced in the medical literature in 1854 by Dr. Richard Quain to describe a mass of unknown origin and consistency incidentally discovered during autopsy [1]. Meticulous studies have since revealed that bezoars constitute compounds of indigestible food residue, which can accumulate and ultimately cause obstructive phenomena in the gastrointestinal (GI) tract. These occurrences have sporadically been reported in the medical literature, accounting for approximately 0.4–4.8% of intestinal obstruction of mechanical etiology [1,2]. Phytobezoars, which comprise plant fiber fragments, are the most common type, accounting for approximately 40% of cases [3]. Herein, we present a case of an elderly male with intestinal obstruction and incipient intussusception owing to multiple phytobezoars located in the ileum.

## 2. Case Presentation

An 81-year-old male presented to the emergency department with intense colicky pain in the right lower quadrant, which had initially appeared the previous day and had since intensified. He also complained of anorexia and bouts of nausea, often followed by bilious vomiting. His temperature was normal, and he did not report diarrhea or constipation. Bowel sounds appeared hyperactive and high-pitched upon auscultation. Physical examination of the abdomen revealed marked sensitivity in the right lower quadrant and hypogastric region, without rebound or guarding. Digital rectal examination was normal. Upon suspicion of a possible intestinal ileus, the patient was admitted to the hospital and started on intravenous (IV) antibiotics while laboratory and imaging tests were ordered. Nil per os was instructed, and a nasogastric (NG) tube was placed in order to decompress the upper GI tract.

Regarding his past medical history, the patient had suffered an acute myocardial infarction (AMI) 15 years prior, for which he had undergone percutaneous coronary intervention (PCI). Ever since, he has been under medication with acetylsalicylic acid and simvastatin, the former of which was discontinued in light of the patient undergoing endoscopy the day after his hospital admission; accordingly, the patient received subcutaneous bemiparin injections once daily for the duration of his hospital stay. He was also under medication with metoprolol in order to regulate his blood pressure. He had undergone laparoscopic cholecystectomy 4 years prior, as well as surgical repair of bilateral inguinal hernias with mesh placement. Owing to the aforementioned previous surgeries, the ileus was attributed to possible postoperative adhesions in the abdominal cavity.

Laboratory tests revealed normal inflammatory markers; white blood cell (WBC) count was 8.44 K/μL (normal range (nr): 4–11 K/μL), and C-reactive protein (CRP) was 0.1 mg/dL (nr: <0.5 mg/dL). Hemoglobin (Hb) was 14.4 g/dL (nr: 13.0–18.0 g/dL) and platelet count was 263 K/μL (nr: 150–400 K/μL). Regarding his renal function, serum urea was 33 mg/dL (nr: 10–50 mg/dL), and serum creatinine was 0.8 mg/dL (nr: 0.7–1.2 mg/dL). Liver enzyme levels were also normal. The patient’s electrolyte count was also examined; serum potassium was 4.5 mmol/L (nr: 3.5–5.1 mmol/L), serum sodium was 141 mmol/L (nr: 136–145 mmol/L), and serum calcium was 10.2 mg/dL (nr: 8.6–10.2 mg/dL). Additionally, INR was 1.18.

**Figure 1:**
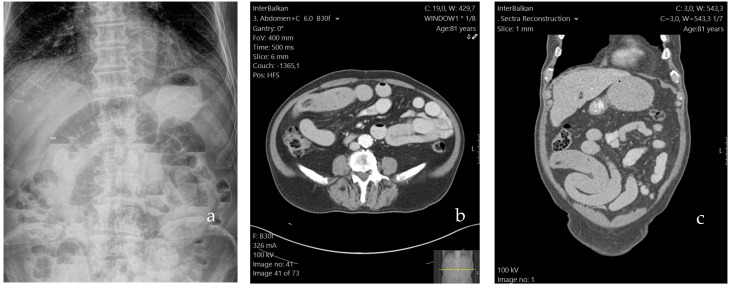
The patient’s preoperative abdominal X-ray (**a**) and CT scan; axial (**b**) and coronal (**c**) plane.

Initial imaging included chest and abdominal X-rays; the former was normal, while the latter revealed the presence of air-fluid levels in dilated loops of the small intestine, therefore confirming the initial diagnosis of an ileus (Figure 1a). The following day, an upper GI endoscopy (esophagogastroduodenoscopy) was performed, revealing mild gastritis of the intermediate region. The campylobacter-like organism (CLO) test was negative. On the same day, the patient underwent a contrast-enhanced abdominal computed tomography (CT) scan in order to decipher the cause of the intestinal obstruction. The results revealed proximally distended loops extending from Treitz’s ligament to the ileum, where a distinct transitional point was observed. In that particular segment, the ileum exhibited concentric thickening, indicating an intraluminal obstructive cause. Bowel loops distal to the obstruction appeared collapsed (Figure 1b,c). Additional findings included pericolic fluid retention, as well as fluid in the pouch of Douglas (POD), with slightly enlarged mesenteric lymph nodes adjacent to the caecum. Taking into consideration the patient’s advanced age, the above imaging findings raised suspicion of a possible malignancy.

Based on the aforementioned radiological imaging, as well as the deterioration of the patient’s clinical status, an emergency laparotomy was performed. Under general anesthesia and through a midline incision, the entirety of the peritoneal cavity was surveyed. Careful investigation of the small bowel revealed multiple palpable, slightly mobile masses approximately 40 cm from the ileocecal junction; the proximal part of the small intestine was visibly distended and intussuscepted. The intraluminal formations were of hard consistency, with an abnormal shape, imitating feces.

**Figure 2:**
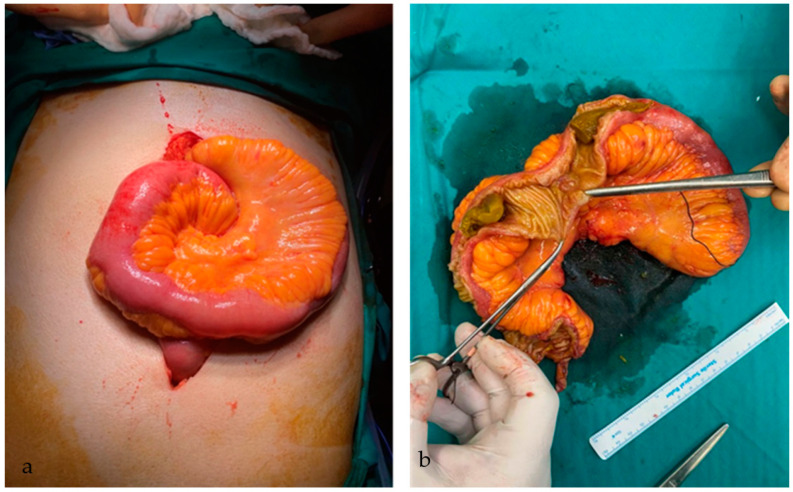
Thickening and extension of the fat as seen intraoperatively, along with impending ischemia of the formerly intussuscepted segment of the small intestine (**a**). The surgical specimen; visible ulceration of the intraluminal wall and two phytobezoars, approximately 5 cm each (**b**).

In the distended segment of the small bowel, visible ulcerations and hypertrophy of the mesenteric fat were also observed. Notably, the thickening and extension of the fat around the circumference of the intestine were similar to “creeping fat”, which is associated with Crohn’s disease (Figure 2a). Owing to the frailty of the intestinal wall and the ischemia resulting from the intussusception, we proceeded with segmental enterectomy 20 cm proximally to the ileocecal valve, with consequent entero-enteric side-to-side anastomosis, using linear surgical staplers. The entirety of the abdominal cavity was consequently rinsed with normal saline, and two drainage tubes were inserted bilaterally in the POD. The patient tolerated the procedure well.

An incision on the antimesenteric border of the surgical specimen revealed the presence of multiple bezoars. Additionally, at the sites of the bezoar’s lodging, the intraluminal wall exhibited prominent ulcerations and strictures (Figure 2b). The entirety of the surgical specimen was subsequently sent for histopathological examination, which disclosed the presence of three mucosal ulcers, which could have facilitated the bezoar’s formation. Inflammatory granulomatous tissue, with purulent cores, was observed peripherally. Plant cellular matter was identified in the extracted bezoars, thus setting the diagnosis of intestinal obstruction due to multiple phytobezoars.

Combined with the aforementioned endoscopy results, it was suspected that our patient’s phytobezoars had formed primarily in the small intestine, owing to alterations in the intraluminal anatomy. The patient’s postoperative recovery period was uneventful; the NG tube was removed on the first postoperative day, and per os diet was initiated. Bowel sounds were normal upon auscultation. He was discharged 6 days postoperatively.

## 3. Discussion

Bezoars constitute a rare medical entity; they exhibit an increased prevalence in advanced ages and tend to affect both genders equally [4]. Bezoars can be primarily classified according to their main ingredient into phytobezoars, lactobezoars, trichobezoars, and pharmacobezoars. Lactobezoars usually occur in premature infants who consume formula milk; they consist of indigested lactose proteins [1]. Trichobezoars comprise hair, and their discovery usually discloses an underlying psychiatric disorder, such as trichotillomania and trichophagia [2]. Pharmacobezoars occur as a result of indigested medications; this is commonly seen in laxatives, slow-released tablets, as well as in medications that have an enteric barrier coating [1,2,5,6]. Phytobezoars constitute the most common type of bezoars; their main compounds are cellulose, hemicellulose, lignin, tannins, and pectins [1,7].

Bezoars can be located in any part of the gastrointestinal (GI) tract, yet the stomach is the most common site [2,7,8]. Bezoars in the small intestine are either primarily formed there or have migrated from the stomach through the pyloric valve [1]. They are usually located in the ileum, owing to the anatomical and physiological features of the region [6]. Colonic, esophageal, rectal, and biliary bezoars are more infrequent [9,10,11,12].

Many studies have been conducted in order to determine predisposing factors for bezoar formation. Generally, it appears that bezoars are usually identified in elderly people and people with reduced gastric or intestinal motility. The most consistent finding in people with phytobezoars is previous gastric surgery, such as vagotomy or partial gastrectomy, which results in decreased gastric motility and hypoacidity [8,13,14,15]. According to a retrospective study conducted by S. Yang and M.J. Cho, these patients have higher postoperative complication rates when surgical intervention to treat phytobezoars is necessitated [13]. Reconstructive surgeries involving the pylorus or the gastric antrum, such as bypass or resection, along with anastomosis, such as a Roux-en-Y, result in anatomical and physiological changes [1,4]. Consequently, large boluses of improperly digested food can advance into the small intestine and cause an obstruction. Systematic reviews also highlight the role of bariatric surgical interventions in bezoar formation [1,4]. Moreover, adhesions from previous abdominal surgeries, radiation therapy, ulcers, strictures, polyps, tumors, and diverticulosis can facilitate primary bezoar formation in the small intestine [5]. Interestingly, bezoars can also be the causative factor for some of the above anatomical alterations through continuous irritation of the intraluminal wall [15].

Systemic conditions and pharmacological agents also have a crucial role in bezoar formation. Hypothyroidism, diabetes mellitus, inflammatory bowel diseases (IBD), malignancies of the GI tract, and neurologic conditions, namely scleroderma and myotonic dystrophy, can also be the instigators of the aforementioned physiological dysfunctions. Moreover, medications such as cholestyramine, anti-acids, anticholinergics, and tricyclic antidepressants can cause gastroparesis [2,6,15]. A distinct subcategory of phytobezoars, seed bezoars, tends to affect people without any predisposing factors; they manifest with different prevailing symptoms and require other forms of treatment, as they are usually located in the rectum [16].

The increased prevalence of phytobezoars in the elderly population can sometimes be attributed to dental problems, which lead to improper mastication and, therefore, non-fragmented food boluses being ingested [4,17]. Their incidence exhibits a notable geographic distribution; most case reports of phytobezoars originate from countries of the Mediterranean [3,14]. This can be attributed to the specific characteristics of that region’s dietary customs, which include foods rich in fiber, such as fruits and vegetables. Additionally, Korea, Japan, and other eastern countries also exhibit an increased prevalence of phytobezoar occurrence; consumption of persimmons, fruits rich in fiber, is common in those areas [2,3].

Clinical manifestation varies according to the bezoar’s location in the GI tract, its composition, and the overall condition of the patient. Asymptomatic cases have been reported, yet in most cases, these medical entities manifest through gastric or intestinal obstruction. Common symptoms include abdominal pain and distension, nausea and vomiting, anorexia and dysphagia, or even constipation [5,14,18]. Endoscopy can accurately visualize, diagnose and even remove a gastric phytobezoar; intestinal phytobezoars constitute a diagnostic challenge. Adhesions from previous abdominal surgeries, benign and malignant tumors of the GI tract, huge polyps, internal hernias, or abdominal cocoon syndrome can cause an ileus, resulting in a similar clinical presentation [19]. It should be noted that in the elderly population, an inconclusive finding suggesting an intraluminal obstructive cause could indicate the presence of a malignant tumor; however, according to phytobezoar’s increased prevalence in that age group, suspicion should be set preoperatively [17].

Imaging tests are essential for diagnosing and differentiating cases of intestinal obstruction. CT appears to be the most advantageous test, especially in setting an early diagnosis of a phytobezoar, with the characteristic “mottled gas pattern” of an intraluminal mass [1,20,21]. Wang et al. have also suggested that an increased CT value could prognosticate the need for surgical intervention [22]. However, in our patient’s case, there was an absence of the aforementioned pathognomonic finding, which has also been reported in the literature; this predicament can hinder setting a definitive preoperative diagnosis [20]. It is useful to consider the additional benefits of a magnetic resonance imaging (MRI) scan in case of an inconclusive CT scan. Contrast-enhanced MRI can more accurately differentiate between phytobezoars and tumors of the GI tract based on their characteristic signal intensity in T1 and T1 weighted images [23].

Bezoar-induced intestinal obstruction may lead to serious complications if left untreated. Gastric or intestinal perforation can be a life-threatening complication, and it requires immediate surgery. Gastric bleeding, intussusception, and strangulation can lead to local ischemia/necrosis [4,13]. Fistula formation, ulcerations, and adhesions are also possible complications, especially in recurrent cases [15]. In rare instances, phytobezoars can spontaneously disappear [2].

Many treatment methods have been utilized in order to treat phytobezoars. If the patient’s clinical status and the phytobezoar’s location do not dictate immediate surgical intervention, conservative methods can primarily be attempted. Gastric phytobezoars are more accessible; metoclopramide can facilitate gastric emptying [4,6]. Dissolution or fragmentation of these conglomerates can be achieved by administering proteolytic enzymes such as papain and cellulase. However, persimmon phytobezoars exhibit a dissolving-refractory type of behavior [1]. Coca-Cola^®^ has also proved to be a reliable first-line treatment for gastric phytobezoars [6,24]. Notably, in an in vitro experiment conducted by Iwamuro et al., where the dissolving efficacy of Coca-Cola^®^ on persimmon bezoar fragments was studied, the well-known beverage achieved significantly better results compared to the enzymatic methods [2]. Nevertheless, it has also been suggested that these residual fragments could subsequently cause obstruction in a distal part of the GI tract and that the enzymes used could cause ulcerations in the esophageal and gastric mucosa [2,4]. 

Recent research articles have also highlighted the efficacy of new methods, such as the transnasal ileus tube (TIT). Compared to a nasogastric (NG) tube, TIT can decompress the entirety of the small intestine proximally to the obstruction and aid in administering enzymes or liquids to dissolve phytobezoars. A research analysis conducted by Lin et al. concluded that conservative methods when combined with TIT, can achieve optimal results and ultimately decrease the need for a surgical approach [25].

Invasive techniques to treat obstruction due to phytobezoars include endoscopic and surgical interventions. Gastric phytobezoars allow for endoscopic fragmentation or removal, which can be highly effective [2,4,13,22]. Obstruction due to intestinal phytobezoar dictates a surgical approach; it is also the method of choice upon the endoscopic treatment’s failure [3,15]. Surgical interventions include enterotomy, the “milking technique”, and even segmental bowel resection. Enterotomy refers to the removal of the phytobezoar through an incision in the small intestine and has proven more successful for proximally located bezoars [1,5,8]. The “milking technique” can be used in various cases; it involves the fragmentation of the phytobezoar into smaller compounds and their subsequent advancement into the colon through the ileocecal valve [4,15]. However, retrospective studies have highlighted an increased rate of complications, such as perforation and formation of postoperative adhesions, in instances where the “milking technique” was used, compared to conventional enterotomy [3].

It is important to note that time is of the essence if any of the above interventions are to be applied. In cases of complete bowel obstruction that result in ischemia, strangulation, or necrosis, segmental bowel resection is the method of choice [1,8,21,26]. In our patient’s case, it was difficult to decipher whether the intraluminal masses revealed on CT were phytobezoars, owing to the lack of the pathognomonic “mottled gas” appearance. The above inconclusive findings, paired with the patient’s advanced age and clinical deterioration, dictated an immediate surgical approach. Taking into consideration the frailty of the intestinal wall and the incipient intussusception, segmental enterectomy and consequent anastomosis was the procedure of choice. In order to prevent a potential recurrence, patients are usually advised to avoid diets rich in fiber. Furthermore, when there is a history of gastric surgery or an underlying systemic condition, are advised to adhere to follow-up guidelines and prescribed medications respectively [27,28].

## 4. Conclusions

Phytobezoars constitute a rare cause of acute intestinal obstruction, and their pathogenesis is multicausal, involving systemic conditions, local factors, as well as dietary customs. Cases of mechanical ileus in the elderly should be viewed carefully; phytobezoar-induced obstruction exhibits an increased prevalence in advanced ages, and early, accurate diagnosis can reduce the need for surgical interventions. CT is the gold standard approach, yet in rare instances, it can produce inconclusive findings; in these cases diagnosis can be set intraoperatively. 

## Data Availability

Not applicable.
